# Community Disaster Resilience: a Systematic Review on Assessment Models and Tools

**DOI:** 10.1371/currents.dis.f224ef8efbdfcf1d508dd0de4d8210ed

**Published:** 2015-04-08

**Authors:** Abbas Ostadtaghizadeh, Ali Ardalan, Douglas Paton, Hossain Jabbari, Hamid Reza Khankeh

**Affiliations:** Department of Disaster Public Health, School of Public Health, Tehran University of Medical Sciences, Tehran, Iran; Department of Disaster Public Health, School of Public Health, Tehran University of Medical Science, Tehran, Iran; Department of Disaster and Emergency Health, National Institute of Health Research, Tehran University of Medical Science, Tehran, Iran; Harvard Humanitarian Initiative, Harvard University, Cambridge, USA; School of Psychological and Clinical Sciences, Charles Darwin University, Darwin, Northern Territory, Australia; Department of Disaster Public Health, School of Public Health, Tehran University of Medical Sciences, Tehran, Iran; Department of Infectious Diseases and Tropical Medicine, School of Medicine, Tehran University of Medical Sciences, Tehran, Iran; Department of Health in Emergency and Disaster, University of Social Welfare and Rehabilitation Sciences, Tehran, Iran; Department of Clinical Science and education, Karolinska Institute, Stockholm, Sweden

## Abstract

Introduction: Recent years have witnessed community disaster resilience becoming one of the most heavily supported and advocated approach to disaster risk management. However, its application has been influenced by the lack of assessment tools. This study reviews studies conducted using the resilience concept and examines the tools, models, and methods adopted. It examines the domains, indicators, and indices have been considered in the tools. It provides a critical analysis of the assessment tools available for evaluating community disaster resilience (CDR). Methods: We investigated international electronic databases including Scopus, MEDLINE through PubMed, ISI Web of Science, Cochrane Library, Cumulative Index to Nursing and Allied Health (CINAHL), and Google Scholar with no limitation on date, and type of articles. The search terms and strategy were as follow: (Disaster* OR Emergenc*) AND (Resilience OR Resilient OR Resiliency) that were applied for titles, abstracts and keywords. Extracted data were analyzed in terms of studied hazards, types of methodology, domains, and indicators of CDR assessment. Results: Of 675 publications initially identified, the final analysis was conducted on 17 full text articles. These studies presented ten models, tools, or indices for CDR assessment. These evinced a diverse set of models with regard to the domains, indicators and the kind of hazard described. Considerable inter dependency between and among domains and indicators also emerged from this analysis. Conclusion: The disparity between the articles using the resilience concept and those that offer some approach to measurement (675 vs. 17) indicates the conceptual and measurement complexity in CDR and the fact that the concept may be being used without regard to how CDR should be operationalized and assessed. Of those that have attempted to assess CDR, the level of conceptual diversity indicates limited agreement about how to operationalize the concept. As a way forward we summarize the models identified in the literature and suggest that, as a starting point for the systematic operationalization of CDR, that existing indicators of community disaster resilience be classified in five domains. These are social, economic, institutional, physical and natural domains. A need to use appropriate and effective methods to quantify and weigh them with regard to their relative contributions to resilience is identified, as is a need to consider how these levels interrelate to influence resilience. Although assessment of disaster resilience especially at the community level will inform disaster risk reduction strategies, attempts to systematically do so are in preliminary phases. Further empirical investigation is needed to develop a operational and measurable CDR model.

## Introduction

Worldwide risk from natural, health, environmental and terrorist hazards is increasing 1 ^,^ 2 . The past two decades has witnessed natural disasters, acts of terrorism, infectious diseases outbreaks, and social unrest that affect more than 200 million people annually 2. Despite the fact that comprehensive disaster management principles advocate prevention (reduction), mitigation, preparedness (readiness), response and recovery issues, the majority of these activities have focused on post disaster (response and recovery) activities^3^.

The lack of attention to prevention or readiness means not only that the demands placed on response and recovery resources are unnecessarily increased, but also people are being placed at greater risk. Recognition of the latter has, in recent years, resulted in the international approach to emergency and disaster management shifting to a more systematic and comprehensive risk management process. This approach has focused attention on developing a more integrated approach to pre-disaster (prevention, mitigation and preparedness actions that are identified as Disaster Risk Reduction activities) and post-disaster activities 3 ^,^ 4.

Pivotal to this new approach has been increased emphasis on enhancing community resilience to reduce impacts of disasters 1 ^,^ 2 ^,^ 3. Community disaster resilience (CDR) has become the cornerstone of hazard readiness and disaster risk reduction in developed countries 5. The National Strategy for Public Health and Medical Preparedness of the United States identiﬁed community resilience as one of the most critical components of public health and medical preparedness. The National Health Security Strategy adopted community resilience as the vision of national health security. The US Department of Homeland Security identifies community resilience as one of the key foundational concepts for security, and the National Disaster Recovery Framework recognizes community resilience as fundamental to successful recovery processes  5.

However, despite the growing importance and interdisciplinary adoption of the resilience construct, no clear definition for this term has emerged 5 ^,^ 6 ^,^ 7 ^,^ 8 ^,^ 9. A systematic review in 2011 suggested that the best definition for resilience is that used by the United Nation International Strategy for Disaster Reduction (UNISDR) 6. UNISDR defines resilience as *“the ability of a system, community or society exposed to hazards to resist, absorb, accommodate to and recover from the effects of a hazard in a timely and efficient manner, including through the preservation and restoration of its essential basic structures and functions*” 10. However, operationalizing this definition presents its own set of challenges.

While it is undeniable that, for example,“resisting” and “absorbing” are universally applicable, they (and the other components embodied in the definition) represent separate processes and how they are enacted varies from hazard to hazard (e.g., flood readiness strategies differ from those required for seismic hazards) and from country to country (e.g. reduction and readiness program in Japan differ from those in New Zealand, yet both face comparable levels of seismic risk). This means that not only is it important to operational resilience, how it is operationalized must be able to encompass, for example, the hazard, cultural and national diversity that prevails in an international context. In addition to developing a robust operational definition, it is important to identify why there are differences between communities with regard to levels of resilience. That is, it is also pertinent to identify the variables and processes that influence or predict resilience.

Problems identifying predictors mirror those for operationalization. Research on predictors has identified roles for diverse variables (that can vary from culture to culture) 8 ^,^ 11. Variables identified include religious affiliation, place of residence (place attachment)^12 ^,spirituality, ethnicity, culture 13, social trust 14 , community education, community empowerment, practice, social networks, familiarity with local services, physical and economic security, economic development, social capital, information and communication, and community competence 8 ^,^ 4 ^,^ 15 are major elements of community disaster resilience. However, the lack of systematic development of an operationalized model of resilience has meant that the analysis these variables has limited utility (i.e., in the absence of robust operational definition of CDR, it is not possible to evaluate the relative importance or utility of predictor variables). Consequently, identifying a set of variables that can be systematically tested is important if we are to develop a set of measures that can serve to assess and compare levels of resilience from community to community 1 ^,^ 16 ^,^ 17, and use this comparative knowledge to identify and develop interventions and evaluate their effectiveness. The assessment of community disaster resilience is also required to pursue the goal of measuring community progress for resilience enhancement over time and to compare different communities in a larger region as well 16.

A few models and tools for assessing community disaster resilience have been developed more than others 18. For example, Kafle developed a method for measuring community resilience capabilities using process and outcome indicators in 43 coastal communities in Indonesia. He emphasized that community resiliency can be measured but each measurement should be both location and hazard-specific 11. This latter point reiterates the issue introduced above regarding theneed for an all-hazards, multi-community and multi-cultural approach. Kafle’s comments introduce how CDR assessment is in its infancy, that more work on quantified metrics, and identifying what should be quantified, is necessary, and a broader range of case studies on the development and testing of metrics is required 19. The present study was designed to determine which tools, models or methods are available for assessment of community disaster resilience. Moreover, we aimed to determine which domains, indicators and indices have been considered in the available tools.

## Methods

**Search strategy and selection criteria:**

The first issue that needed to be dealt with when researching community resilience is considering what is meant by “community”. Although several definitions for community exist 5, in present study, community was defined as “*A group of people with diverse characteristics who are linked by social ties, share common perspectives, and engage in joint action in geographical locations or setting*” 20. Consequently, the analysis presented in this paper included every geographical– based population living in urban or rural areas including villages, neighborhoods, towns, districts, regions or a part of a mega city.

We investigated international electronic databases including Scopus, MEDLINE through PubMed, ISI Web of Science, Cochrane Library, Cumulative Index to Nursing and Allied Health (CINAHL) and Google Scholar with no limitation on date and type of articles. Using multiple and different words like community, disaster, resilience and assessment as search strategy might be limited potential citations. So, in order to increase the study sensitivity, we used only two main words.

The key terms and search strategy were as follow: (Disaster* OR Emergenc*) AND (Resilience OR Resilient OR Resiliency) that were applied in titles, abstracts, and keywords. Articles related to a community or population and those describe or present a model, tool or index for assessment of community disaster resilience were included in the study. While CDR is a multi dimensional concept, articles merely related to individual, psychological, natural, physical and economic resilience were excluded.

**Data extraction and analysis:**

Data extraction was carried out by two independent researchers. Two data collection forms were developed, and after piloting were used for data extraction. The first one included general characteristics of articles including model or index name, year of publication, study location ( where the model or tool has been developed or implemented), hazard type, methodology used for index or tool development (how the model or tool has been developed), domains and number of indicators. The second form was used for identifying details of domains and indicators in each model, tool, or index. According to the study objectives extracted data were analyzed in terms of hazards approach, methodology of tool or model development, domains and indicators evaluated in each model, and qualitative or quantitative assessment of disaster resilience.

## Results

In the first step, investigation of international electronic database from 7th to 10th September 2013 identified 2,506 potentially relevant citations. In addition, hand reviewing of Google Scholar yielded 180 more potentially citations. Totally, we found 2,686 potentially relevant citations.

After that we discarded 1,333 duplicated citations, we had 1,353 unique and potentially relevant citations. In the next step we reviewed all abstracts and excluded 678 citations on the grounds of their not being related to the community. This yielded 675 community-related potentially relevant citations. Then we studied abstracts in term of describing or presenting tools, models or methods for assessment of community disaster resilience. The lack of coverage of tools and assessment methods led to 637 of 675 citations (94.4 percent) being excluded and 38 potentially relevant citations (5.6 percent) being included for full text reviewing (this provides an indication of the scale of the problem of assessing resilience). Figure 1 shows the PRISMA flow diagram for the identification of studies for this review.

**The PRISMA flow diagram for the identification of studies for this review.**
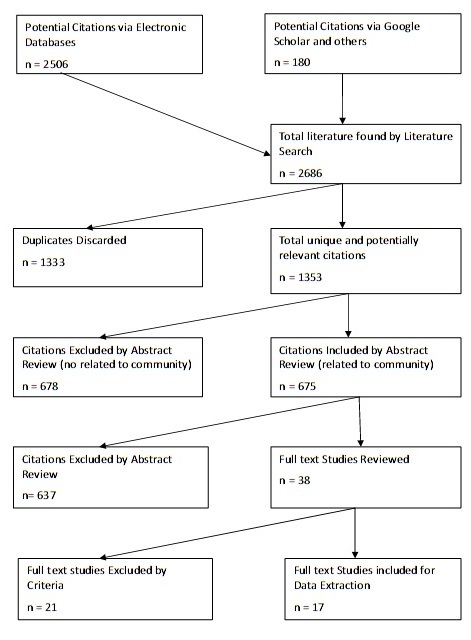

Two reviewers separately evaluated the full texts. In this step we excluded 21 full text articles due to following reasons. 14 articles 5 ^,^ 16 ^,^ 17 ^,^ 20 ^,^ 21 ^,^ 22 ^,^ 23 ^,^ 24 ^,^ 25 ^,^ 26 ^,^ 27 ^,^ 28 ^,^ 29 ^,^ 30 were about community disaster resilience frameworks, actions, planning, characteristics and indicators that present no disaster resilience assessment tool or model. Three articles 1 ^,^ 31 ^,^ 32 were not original and peer review articles. They were guidelines or reports related to disaster resilience. Two articles 19 ^,^ 33 were related to physical and infrastructural resilience. One article 34 measured the level of educational resilience in schools. One article 35 contained no details of community disaster resilience indicators and how they were measured. The application of this strategy produced 17 full text articles that were included in the study. Within these 17 articles, data extraction identified 10 models, tools or index that had been used for community disaster resilience assessment. Table 1 shows general characteristics of these tools or models and their related articles.

**Figure:**
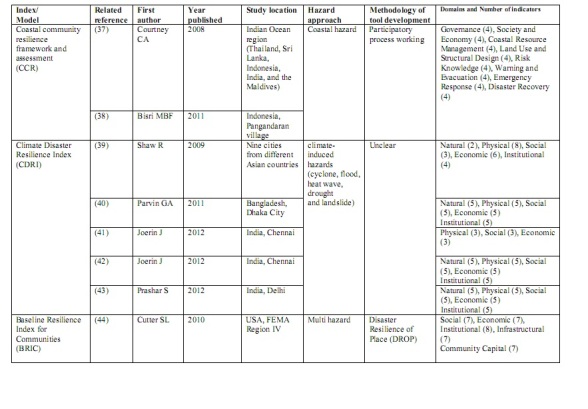

**Figure:**
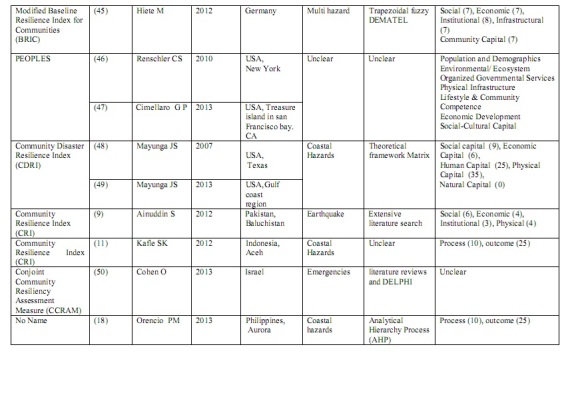
**Table 1: Characteristics of tools or models of community disaster resilience assessment based on included articles (n=17)**

Analysis of the indicators used to predict community disaster resilience in these studies resulted in using only three of ten models in this part of the study. The main reason for doing so was finding a degree of similarity in the indicators used across the models. Consequently, the analysis process was developed around the original and most complete model. Three models used the same indicators 9 ^,^ 36 ^,^ 37. So we used the original article 37. For Climate Disaster Resilience Index 38 ^,^ 39 ^,^ 40 ^,^ 41 ^,^ 42, we used the article which authored by Joerin 39. Three models 11 ^,^ 18 ^,^ 43 assessed process and outcome aspects of disaster resilience. However, they did not identify indicators. Notwithstanding, we considered these models in summarization of the indicators. Two models the PEOPLES and CCRAM 44 ^,^ 45 contained unclear indicators.

The results are summarized in Table 2 which lists the factors, criteria or variables that were clearly identified as predictors of community disaster resilience in models analyzed 37 ^,^ 39 ^,^ 46. An important finding in these studies is their identifying disaster resilience as a multi-level phenomenon.

**Table 2: Domains and indicators used for assessment of community disaster resilience in the studied articles:** 

**Baseline Resilience Index for Communities (BRIC) ** 44 ****	
**Social**	Educational equity; age; transportation access; communication capacity;language competency; special needs for disables; health coverage****
**Economic**	Housing capital, Employment, Income and Equality, Single sector employment dependence, Female Employment, Business size, Health Access****
**Institutional**	Mitigation plan, Flood coverage , Municipal Services, Political Fragmentation, Previous disaster experience, Mitigation (social connectivity), Mitigation (participation), Mitigation( Storm ready)****
**Infrastructural**	Housing type, Shelter capacity, Medical capacity, Access/ evacuation potential, Housing age, Sheltering needs, Recovery****
**Community Capital**	Migration, Place residency, Political engagement, Social capital (religion), Social capital (civic involvement), Social capital (advocacy), Innovation****
**Climate Disaster Resilience Index (CDRI)** 42	
**Social**	Population, Health, Education and awareness, Social capital, Communitypreparedness****
**Economic**	Employment, Finance and savings, Budget and subsidy, Income, Householdassets****
**Institutional**	Mainstreaming of disaster risk reduction, Effectiveness of zone’s crisis management framework, Knowledge dissemination and management Institutional collaboration, Good governance****
**Physical**	Electricity, Water, Sanitation and solid waste, Accessibility of roads,Housing and land use****
**Natural**	Ecosystem services, Land-use in natural terms, Environmental policies Intensity/severity of natural hazards, Frequency of natural hazards****
**Community Disaster Resilience Index (CDRI) ** 49	
**Social**	Nonprofit organizations registered, Recreational and sport centers, Registered voters , Civic and political organizations, Census response rates Religious organizations Owner-occupied housing units, Professional organizations, Business organizations****
**Economic**	Per capita income , Household income, Employed civilian population , Owner-occupied housing units, Business establishments, Population with health insurance,
**Human**	Population with more than high school education, Physicians, Health care support workers, Building construction workers, Heavy and civil engineering construction workers, Architecture and engineering workers, Environmental consulting workers, Environment and conservation workers, Land subdivision workers, Building inspectors, Landscape architects and planners, Property and causality insurance workers, Highway, street, and bridge construction workers, Population employed in legal services, Percentage of population covered by comprehensive plan, Percentage of population covered by zoning regulations, Percentage of population covered by building codes, Percentage of population covered by FEMA approved mitigation plan, Community rating system, Fire fighters, prevention, and law enforcement workers, Population employed in scientific research and development services, Colleges, universities, and professional schools employees, Population that speaks English language very well, Population employed in special need transportation services, Community and social workers
**Physical**	Building construction establishments, Heavy and civil engineering construction establishments, Highway, street, and bridge construction establishments, Architecture and engineering establishments , Land subdivision establishments, Legal services establishments, Property and causality insurance establishments, Building inspection establishments, Landscape architecture and planning establishments, Environmental consulting establishments, Environment and conservation establishments, Scientific research and development establishments, Colleges, Universities, and Professional schools, Housing units, Vacant housing units, Hospitals, Hospital beds, Ambulances, Fire stations, Nursing homes, Hotels and motels, Occupied housing units with vehicle available, Special need transportation services, School and employee buses, Owner-occupied housing units with telephone service, Newspaper publishers, Radio stations, Television broadcasting, Internet service providers, Temporary shelters, Community housing , Community food service facilities, Schools, Licensed child care facilities, Utility systems construction establishments

Another issue that emerged from the comparative analysis of the articles was considerable diversity in definition of and use of the words used to describe “domain,”“indicator” and “index”. Words such as domain, component and dimension had the same meaning in different models. In addition, words such as indicator, factor, variable and criteria had been used for factors that impacted directly on the level of community disaster resilience. Furthermore, index and composite indicator had been used for the final number or level of the assessment 9 ^,^ 37 ^,^ 46. Most models and tools had used the term “Index” for final representative of the community disaster resilience.

As Table 1 shows, there were a variety of domains and indicators used for community disaster resilience assessment. The number of domains varied from two to eight. Disparity on the range of indicators used was also evident. Some domains have the same meaning, and some are integrated or interdependent with others e.g., structural and physical domain). This study showed that similar indicators (variables) have used in different domains. For instance as Table 2 shows in Baseline Resilience Index for Communities (BRIC) 37, health access has been used in social, economic and institutional domains. So, this table shows that there is a wide Inter dependency between and among the dimensions and indicators.

## Discussion

There was a considerable disparity between the number of papers that included an identifiable reference to “community resilience” and those that actually attempted to measure community resilience (675 vs. 17 respectively). This disparity provides a tangible indication of the proliferation in the use of the concept of “community resilience,” the limited attention paid to its definition and systematic study, and the consequent need to identify a set of predictors that can inform the systematic assessment process.

The selection process for articles used in this study reveal the dearth of studies that specifically attend to issues of the measurement of and operationalizing of resilience. The lack of agreement on how the resilience concept translates into a measurable framework creates problems not only with regard to the practical implementation of resilience within at-risk communities, but also for systematic research and the development of policy. A shared or common definition of the concept and how it can be measured is required to provide the foundation for the development of the concept and to guide research. This study thus identifies a need to focus on developing a common conceptual and measurement model and that doing so is a precursor to how it can be systematically studied and developed. It was also apparent that the selection of domains and their constituent variables lacked a coherent theoretical foundation. Each model developed in theoretical or conceptual isolation from the others. Thus while there is some overlap in domains/content, differences are also present. For example, the social domain in the BRIC and Climate-DRI, but this is excluded from the Community-DRI (Table 2). However, there is no overlap with regard to the other variables.

The models can also be differentiated with regard to the domain names and how variables are distributed between them. They also mix demographic (e.g., education, disability) and structural characteristics (land use, housing type) with social and psychological (e.g., social capital) characteristics. The diversity evident in Table 2 highlights a need for the development of a common conceptual or theoretical framework from which systematic study can develop. In the absence of the latter, the confusion over the use of the term and how it is assessed and developed will continue. While additional work needs to be directed to the latter, social capital and empowerment concepts represent overarching theoretical constructs that could be used in this context.

While the identification of the diverse domains in which resilience characteristics are situated (e.g., social vs. infrastructure vs. economic) correctly identifies the multi-level nature of the phenomenon, this raises additional issues around measurement and change. Operationalizing factors such as education level or housing type is relatively straightforward. More work has to be done to develop valid and reliable measures of social capital that can be used across cultures and for diverse hazards. The models can also be criticized for the lack of inclusion of specific social and psychological factors (e.g., self- and collective-efficacy, sense of community) that have been empirically demonstrated to influence adaptation.

The models are also lacking in attempts to quantify the relationships and inter dependencies between variables and particularly between levels of analysis. For example, in the BRIC it could be hypothesized that levels of education and communication capacity could predict levels of political engagement, civic involvement and advocacy. If this relationship exists, it could be interpreted to mean that only political engagement etc, are predictors of resilience (i.e., the ability to cope and adapt to novel disaster conditions) and that education represents a mainstream resource in which resilience characteristics are developed.

The latter point also raises issues regarding the development of resilience.The majority of the characteristics identified in these models can only be developed and changed through social policy and political change. This undermines principles of contemporary DRR that highlight the importance of shared responsibility as a basis for resilient communities.

This study showed the lack of comparable conceptualizations regarding the domains, variables and indicators associated with the use of the resilience concept. Using several words for a similar concept could be confusing for readers and researchers alike. Although some scientists might disagree with the following words, we suggest the word domain instead of similar words like component and dimension, as well as indicator for factor, variable, criteria and also index for composite indicator. Since factor or variable was identified as a circumstance, fact or influence that contributes to a result, and criterion or indicator was defined as a quantitative or qualitative measure that simply shows the reality of a complex situation 36 ^,^ 37, we could name all of them as indicator. In addition, because of most models had been used the word of index as final number or level of the community disaster resilience, we also suggest this word instead of composite indicator. An index is the mathematical combination of several indicators that each one is the representative of a special situation 36 ^,^ 37.

Despite different classification of community disaster resilience domains, we can categorize them in five domains including social, economic, institutional, physical and natural. We summarize these domains and their synonyms or sub categories in Table 3. While the identification of multiple levels and domains highlights the need for developing a composite index, as yet no studies have systematically quantified the relationships and inter dependencies that bind levels together and no studies have attempted to quantify the relative weightings (or relative contributions) of the predictors or levels. Nor have they taken into account the fact that different predictors play different roles (different relative weightings) at different times as people and communities transition through different stages of response and recovery that span a period of months or even years 53.

Complying with the latter recommendation is complicated by the infrequent nature of large-scale hazard events and the fact that most studies are based on analysis of community characteristics rather on whether, how and what ways do these characteristics inform understanding of how community members anticipate (preparation/readiness), cope with, adapt to, recover from and develop from experience of disaster. This does not negate the potential value the above analyses, but it does call for more work on identifying how they actually influence resilience.

**Table 3: Domains and their synonyms or sub-categories of community disaster resilience:** 

Domain	Synonyms or sub-categories
**Social **	Human Capital, Lifestyle and Community Competence, Society and Economy, Community Capital, Social and Cultural Capital, Population and Demographics Environmental, Risk Knowledge
**Economic **	Economic Development, Society and Economy
**Institutional**	Governance, Organized Governmental Services, Coastal Resource Management, Warning and Evacuation, Emergency Response, Disaster Recovery
**Physical **	Physical Infrastructure, Infrastructural , Land Use and Structural Design
**Natural**	Ecosystem

In addition, we tried to summarize indicators that used in different models based on the five domains, but the variety in terms of domain classification, definitions and concept makes this problematic. This reiterates the need for international attention to be directed to developing a common framework that can be used as a foundation for the systematic and rigorous development of the resilience concept. However, before this list can be used to inform research and the systematic assessment of resilience, additional work is required to operationalize them. In addition to developing valid and reliable measures, the cross cultural utility of the variables needs to be established. This may require identifying generic predictors that can be translated for use within a specific culture.

Despite some models identifying an ecological (natural) domain as one of the main domains of disaster resilience, this domain had been excluded from the assessment due to data inconsistency and questions regarding its relevancy 37 ^,^ 46. We need to define natural disaster resilience indicators and a model for prioritization and measuring them.

Wide inter dependency of dimensions and indicators makes the process of community disaster resilience assessment some difficult and inaccurate. It seems that using a combination of fuzzy Delphi method (FDM) and Analytic Network Process (ANP) technique like the method developed by Chan 28 or Trapezoidal fuzzy DEMATEL method 36 could help solving this problem. These methods are systemic and effective solution to quantify and weigh interdependent and multiple domains and indicators.

We can classify the models of community disaster resilience in two groups. The first group measures the level of disaster resilience based on existing status of disaster resilience characteristics 37 ^,^ 46. The second group has a process and outcome approach that measures both the process and the outcome of actions and programs which could be increased the level of disaster resilience in the community 11 ^,^ 18 ^,^ 39 ^,^ 47 ^,^ 48. While disaster resilience processes and outcomes are developed and implemented based on the basic indicators, it seems that the first group could assess the level of community disaster resilience rather than the second group.

This study showed that most methods are predominately qualitative 11 ^,^ 18 ^,^ 39 ^,^ 41 ^,^ 42 ^,^ 43 ^,^ 44. Although it might be because of the qualitative nature of resilience indicators 16, we have to develop quantitative and more accurate models.

The use of accurate data is another challenge in CDR assessment. Our study showed that quantitative models and those were developed through a high quality study have used national or regional secondary data for county or community level 37 ^,^ 49 National data are often out of date and inadequate for local level 44. Although local data are not always available, community disaster resilience assessment needs local- or community-level data 16 ^,^ 37.

None of current models has assessed spatial indicators of community disaster resilience such as elevation. Spatial and temporal indicators (place - specific) are indicators that should be included in community disaster resilience assessment 16.

## Conclusions

Present study showed that there are at least five defined and measurable domains for community disaster resilience including social, economic, institutional, physical, and natural. While community disaster resilience is a culture bound concept and also related to the kind of hazards any attempt for assessment should be based on both location and hazard.This study showed that the community disaster indicators differ from a community to another one. So, it seems that the first step for community disaster resilience is determining community resilience indicators.

The disparity between the articles using the resilience concept and those that offer some approach to measurement (675 vs. 17) indicates the conceptual and measurement complexity in CDR and the fact that the concept may be being used without regard to how CDR should be operationalized and assessed. Of those that have attempted to assess CDR, the level of conceptual diversity indicates limited agreement about how to operationalize the concept. As a way forward we summarize the models identified in the literature and suggest that, as a starting point for the systematic operationalization of CDR, that existing indicators of community disaster resilience be classified in five domains. These are social, economic, institutional, physical and natural domains. A need to use appropriate and effective methods to quantify and weigh them with regard to their relative contributions to resilience is identified, as is a need to consider how these levels interrelate to influence resilience. Although assessment of disaster resilience especially at the community level will inform disaster risk reduction strategies, attempts to systematically do so are in preliminary phases. Further empirical investigation is needed to develop a operational and measurable CDR model.

Regarding inter dependency between and among the dimensions and indicators of community disaster resilience, we need to use appropriate and effective methods to quantify and weigh them with regard to their relative contributions to resilience (and to assess show weightings change spatially and over time).Regarding on adjustment of use of data and the level of intervention, we need to incorporate the community data for local decision making. Although assessment of disaster resilience in national and regional level would be useful, local and community disaster resilience measurement is more appropriate for disaster risk reduction and management.

## Limitations

Only English articles were included in this systematic review.

## Correspondence

Ali Ardalan. Department of Disaster & Emergency Health, National Institute of Health Research, Tehran University of Medical Sciences, Tehran, Iran. Email: aardalan@tums.ac.ir

## Authors contributions

Abbas Ostadtaghizadeh, Ali Ardalan and Douglas Paton have contributed in study design, acquisition of data, data analysis, drafting the article, and development of the final manuscript. Hossain Jabbari and Hamidreza Khankeh have critically evaluated the draft article. All authors reviewed and approved the final version of the manuscript.

## Appendix 1

### PRISMA CHECKLIST

PDF
